# Integrating yoga into anatomy and clinical medicine education: A holistic approach to learning

**DOI:** 10.1002/ase.70225

**Published:** 2026-03-19

**Authors:** Dana Rohde, Madeleine E. Norris, Jennifer R. Kinder, Barbie Klein, Derek Harmon

**Affiliations:** ^1^ Department of Anatomy University of California, San Francisco School of Medicine San Francisco California USA; ^2^ Department of Physiology University of California, San Francisco School of Medicine San Francisco California USA; ^3^ Department of Medical Education University of Miami, Miller School of Medicine Miami Florida USA; ^4^ Department of Physical Therapy and Rehabilitation Science University of California, San Francisco School of Medicine San Francisco California USA; ^5^ Department of Cell and Tissue Biology University of California, San Francisco School of Dentistry San Francisco California USA; ^6^ Department of Biomedical Education and Anatomy The Ohio State University College of Medicine Columbus Ohio USA

**Keywords:** anatomy, experiential learning, holistic, musculoskeletal, neuroanatomy, neurological exam, peripheral and spinal nerves, yoga

## Abstract

Anatomical knowledge is fundamental for success in clinical settings. Unfortunately, anatomy education within professional health programs has experienced a continual decrease in contact hours and curricular content over the previous two decades, leading to deficits and potential gaps in anatomical science knowledge. Additionally, there is a common misconception among students that anatomy education is simply a collection of facts that require memorization. Thus, there is a need for innovative teaching methods to help reinforce such knowledge and support students' wellness. This report outlines a series of novel yoga‐based workshops in anatomy education with clinical medicine applications, embodied learning, and a holistic pedagogical approach which engages the mind, body, and spirit. Three yoga workshops focused on (i) musculoskeletal anatomy, (ii) spinal and peripheral nerves, and (iii) neuroanatomy and the neurological exam were developed. Each workshop included a warmup, sun salutations (a series of yoga poses performed in a continuous sequence), *asanas* (individual poses), a *vinyasa* (flow of poses), a cooldown, *savasana* (resting pose), and a closing meditation. The workshops also examined the etymology of Sanskrit and Latin that underpin anatomical and medical terminology. The workshops have been taught to medical students, physical therapy students, and medical residents. A pretest was administered before each session, while a posttest and wellness survey were distributed following the yoga workshops. These workshops offer a template for educators to provide an alternative approach to learners by offering a yoga session that reinforces anatomical knowledge, highlights relevant clinical correlates, and improves overall well‐being.

## INTRODUCTION

Students often approach gross anatomy through rote memorization rather than conceptual understanding, relying on techniques, such as highlighting, creating flowcharts, or crafting stories.^1^ However, anatomical facts in isolation can be overwhelming and lack relevance when they are not linked to a clinical context. In addition, the proliferative amount of content and demanding schedules of medical school curricula may cause high levels of stress and anxiety in medical students.^2^ Thus, there is a need for innovative learning methods to foster students' anatomical science knowledge acquisition and retention, in addition to promoting overall wellness.

### Experiential learning and yoga anatomy studies

Experiential learning is the process by which knowledge is created through experience, and it has been shown to decrease students' reliance on memorization.^3^ The practice of yoga can be a form of experiential learning, with many previous studies integrating yoga and anatomy.^4^, ^5^, ^6^, ^7^, ^8^, ^9^, ^10^

Numerous prior studies have incorporated yoga with anatomy and have demonstrated positive results on both anatomy comprehension and learner wellness. Some previous studies were aimed at yoga practitioners^5^ or undergraduate students,^7^ but most yoga anatomy studies have focused on medical students.^4^, ^6^, ^8^, ^9^, ^10^ When yoga was used as an experiential learning tool, medical^4^, ^6^ and undergraduate^7^ students demonstrated a significant improvement in their comprehension of musculoskeletal anatomy. Furthermore, in addition to being experiential, combining yoga with anatomy education engages kinesthetic learning. Previous studies have highlighted the benefits of kinesthetic learning in anatomy education.^11^, ^12^ Yoga has also been shown to enhance medical student wellness by decreasing stress levels whether anatomy was incorporated^4^, ^8^ or as a stand‐alone activity.^13^

The methodologies of previous studies that integrated yoga and anatomy have been variable. Some studies interspersed individual yoga poses with short anatomy didactics and worksheets,^7^ while others focused on a specific region or regions of the body.^4^, ^5^, ^6^ Feedback from previous research demonstrated that students preferred that they spend more time in the poses and that the yoga was taught concurrent, not intermittently, with the anatomy discussions.^4^, ^7^ One study, focused on first‐year medical students, used yoga as a didactic tool for musculoskeletal anatomy through short videos focused on bones, muscles, and joints, but did not accentuate the holistic aspects of yoga, which focuses on the mind, breathing, and the spiritual aspect.^10^ Another study held yoga sessions (led by a certified yoga teacher) focused only on the anatomy topics for that week.^9^ While there were no differences in test scores on anatomy exams, there was a decrease in self‐reported stress scores immediately after each session for the yoga group compared with the control group.^9^

### Holistic education and embodied learning

Holistic education includes experiential learning with a significant emphasis on the relationship between the mind, body, and spirit.^14^ Additionally, experiential learning is concerned with an individual's social, physical, emotional, artistic, and creative potentials.^14^, ^15^, ^16^, ^17^ A holistic pedagogy encompasses balance (the individual is viewed as a whole rather than the sum of its parts), connection (relationship between the mind and the body), and inclusion (to create the best learning environment for diverse student populations).^15^ This contrasts with the prevailing approach to contemporary education, which is a Cartesian dualism of mind and body where recess, emotions, feelings, movement, craftsmanship, and hands‐on learning have been diminished.^15^

The aim of this curricular approach was to create a series of holistic yoga workshops that incorporate anatomy. Unlike some of the earlier studies with yoga poses interrupted by anatomy didactics,^4^, ^7^ these workshops simulate a full yoga class with breathing exercises, a warmup, sun salutations, *asanas* (poses), *vinyasas* (flows that unite the poses), a cooldown, and a final *savasana* (corpse pose). Anatomical concepts are reinforced concurrently throughout the yoga sessions, rather than intermittently. These workshops balance anatomy and clinical applications with movement and target the entire musculoskeletal system, including the head, neck, abdomen, back, and limbs, as well as the central and peripheral nervous system. In addition, these workshops include a spiritual component incorporating *mudras* (ritual hand gestures in Hinduism and Buddhism) and an explanation of the final chant, *Om*. Sanskrit terminology is utilized with a link to relevant anatomical terms and inclusive artwork is incorporated throughout. Linking the mind and body through action and engaging the whole person intellectually and physically creates a holistic approach with embodied learning.^14^, ^15^

## DESCRIPTION

*Yoga with Danatomy* workshops were designed to help health professions education students and medical residents review and reinforce in‐depth anatomical and clinical knowledge through yoga, while also promoting their overall well‐being. The research team met with clinicians from various departments (anesthesiology, neurology, orthopedic surgery, and physical therapy) to identify high‐yield clinically relevant anatomical concepts. For example, the myotomes presented correlate with those used by neurologists and are common in their clinical practice. Using the identified anatomical and clinical topics as a guide, three unique yoga workshops were created: (i) Musculoskeletal, (ii) Spinal and Peripheral Nerves, and (iii) Neuroanatomy and the Neurological Exam.

### Yoga icon and mantra

**FIGURE 1:**
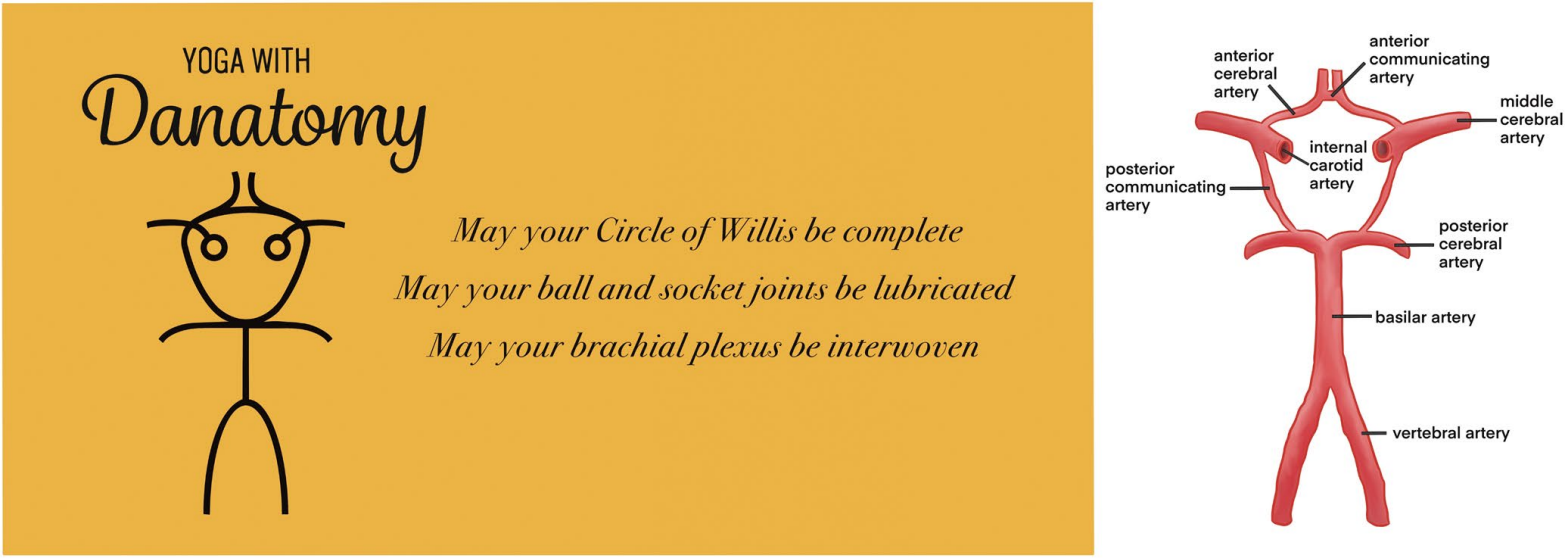
*Yoga with Danatomy* icon and mantra. The icon is based on the cerebral arterial circle (of Willis). The mantra highlights neurovasculature, joints, and peripheral nerves. (Drawing of arteries by Dana Rohde.)

The yoga icon for these workshops is an interpretation of the cerebral arterial circle (of Willis), the arteries at the base of the brain. The mantra focuses on neurovasculature, the musculoskeletal system, and the peripheral nervous system (Figure 1). The cerebral arterial circle is a good starting point for discussions about anatomical variations as well as the importance of collateral circulation. Joints are highlighted as much of yoga focuses on the musculoskeletal system. Finally, the interwoven nature of the brachial plexus is emphasized to recognize the clinical importance of spinal nerve contributions to the terminal branches of the brachial plexus.

### Overview of the workshops

The workshops include a student version and an instructor version of the PowerPoint slides (Microsoft, Redmond, WA, Version 16.104.1, 2025); the former was presented on screens during the workshop. A pretest, posttest, and a one‐question wellness survey were created to assess each workshop. The pretests and posttests were reviewed by the authors and edited based on feedback from students who participated in early pilot studies of the workshops (prior to data collection). The pretest was administered immediately before each workshop, while the posttest and wellness survey were distributed immediately after each yoga session. Both the pretests and posttests consisted of 12 comparable (in content) multiple choice questions, albeit not identical. Ten of the questions covered content presented in the yoga session, while two control questions covered content that the attendees learned in their previous anatomy courses, but were not explicitly reviewed in the yoga session. The wellness survey consisted of one question rated on a 1–5 Likert scale.

From 2019 to 2025, 24 workshops were offered to health professions students and medical residents on campus. The participants were provided with yoga mats and blocks. Each workshop began with learners completing the pretest assessment, followed by breathing exercises and a series of warmup poses. Next, the participants were led through *Surya Namaskar A* (sun salutation A) with a focus on tying breath to movement. Participants were then taught multiple *asanas* (poses) with detailed instructions including suggestions for modifications. The specific *asanas* were selected to demonstrate a wide variety of muscle actions, innervations, and/or clinical concepts. Subsequently, the *asanas* were linked together into a *vinyasa* (a sequence that flows). This was followed by *Surya Namaskar B* (sun salutation B), which includes more advanced asanas, and then a cooldown and *savasana* (final resting ‘corpse’ pose). The yoga session closed with a final meditation, and the chant *Om*—a sacred sound, a spiritual icon, and a mantra. Finally, to conclude, participants completed the posttest assessment, a wellness survey, and an evaluation of the workshop. Each workshop (including the pretest, posttest, and survey) lasted ~90 min. A summary of the workshop's organization is shown below.

- Pretest
- Warmup
- Sun salutation A
- *Asanas* (poses)
- *Vinyasa* (flow)
- Sun salutation B
- Cooldown
- *Savasana* (corpse pose)
- *Om* (final chant)
- Posttest
- Wellness survey
- Evaluation

The meaning of the *Om* symbol was explained at the conclusion of the workshops as a sound in visual form and chanted as three syllables (A‐U‐M), which represent several important triads: earth, atmosphere, and heaven; thought, speech, and action; birth, life, and death; body, mind, and soul. It was also explained that while chanting, one can feel the three syllables of *Om* anatomically, as the reverberations move through the abdomen, thorax, and finally the mouth.^18^

The artwork in these workshops was all custom‐created, hand‐painted watercolors and drawings by two medical students at the University of California, San Francisco. The artists' rendering of the *asanas* used in the workshops is inclusive, based on the diverse population of the medical school class.

### Clinical application

While the aforementioned yoga anatomy studies focused on the musculoskeletal system, these workshops included innervations and clinical concepts. The Musculoskeletal Workshop focused on muscles, their actions, and innervation by peripheral nerves. The Spinal and Peripheral Nerves Workshop added spinal nerves (myotomes and dermatomes) and concentrated on distinguishing between radiculopathies and peripheral neuropathies. The Neuroanatomy and Neurological Exam Workshop reviewed key neuroanatomy concepts. It also covered all parts of the neurological exam, progressing through the structured order: mental status, cranial nerves, motor, reflexes, sensory, coordination, and gait.

### The Musculoskeletal Workshop

**FIGURE 2:**
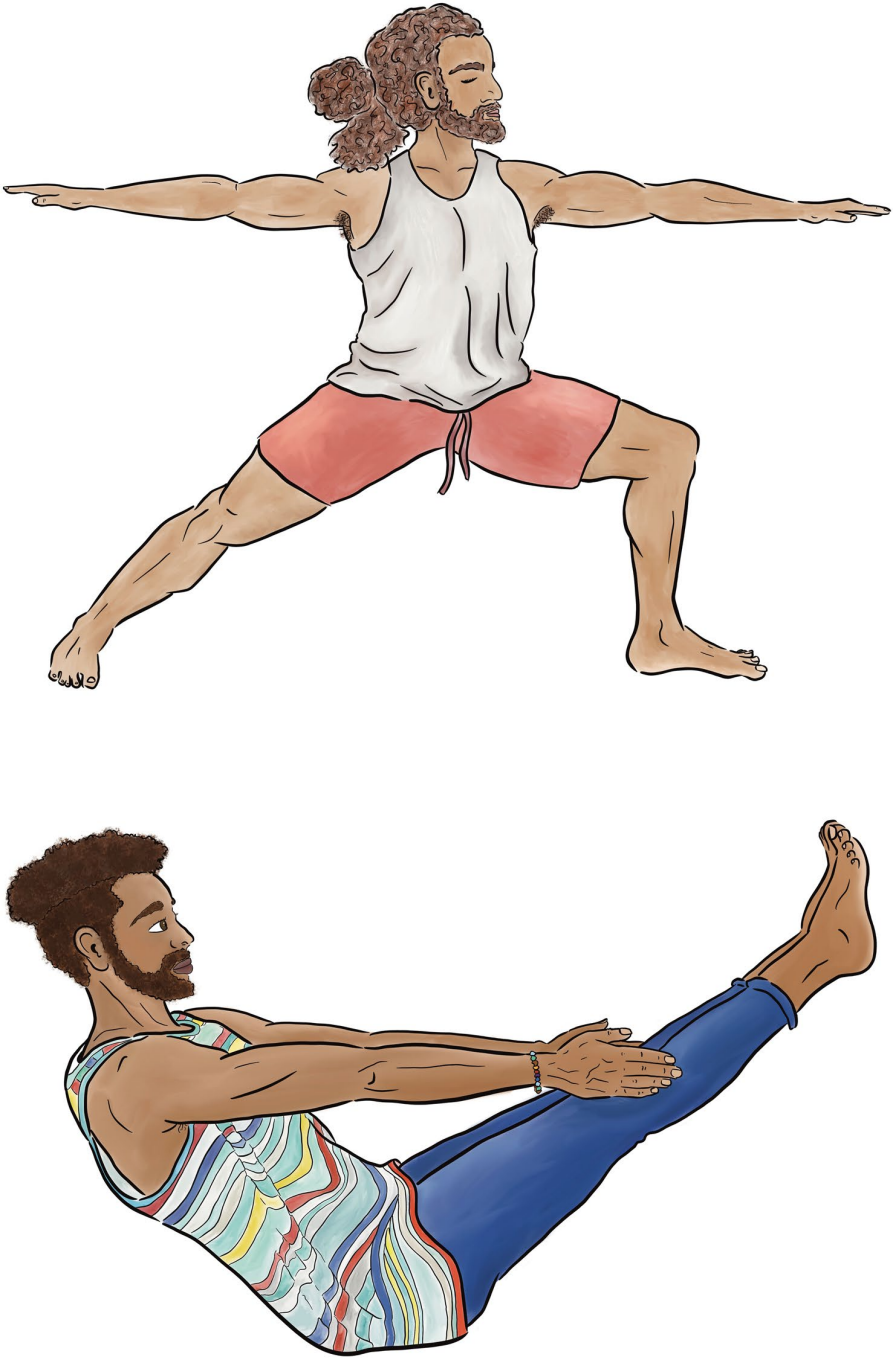
Student artwork showcasing *asanas* that demonstrate muscles in the head, neck, upper limb, trunk, and lower limb. Tables with select muscles, actions, and innervations accompany the drawings in the projected slides. Top panel: *Virabhadrasana II* (warrior II pose); Bottom panel: *Navasana* (boat pose).

Table 1 lists the *asanas* in the Musculoskeletal Workshop, along with the highlighted muscles, their actions, and innervations. For each *asana*, we focused on just a few muscles used to attain the pose, ensuring that a wide array of muscles was covered throughout the workshop. For example, head and neck muscles are highlighted during head rotation in *Virabhadrasana II* (warrior II pose), while limb and trunk muscles are highlighted in *Navasana* (boat pose) (Figure 2). Specific muscles, actions, and innervations are included in tables that accompany these drawings in the projected slides. After teaching the individual *asanas* and linking them in a *vinyasa*, the attendees are asked to recall the muscles, actions, and innervations while assuming the postures.

**TABLE 1:** *Asanas* and select muscles, actions and innervations presented in the Musculoskeletal Workshop.

*Asana* (pose)	Action	Muscles	Peripheral nerves
*Utkatasana* (Chair)	Hip flexion	Iliopsoas	Femoral
	Knee flexion	Hamstrings	Sciatic
*Virabhadrasana I* (Warrior I)	Neck extension	Semispinalis capitis	Dorsal rami
		Splenius capitis	Dorsal rami
	Back extension	Erector spinae	Dorsal rami
*Virabhadrasana II* (Warrior II)	Head rotation	Sternocleidomastoid (contralateral)	CN XI
		Splenius capitis (ipsilateral)	Dorsal rami
	Forearm pronation	Pronator teres and pronator quadratus	Median
*Utthita trikonasana* (Extended triangle)	Shoulder abduction	Deltoid	Axillary
	Knee extension	Quadriceps femoris	Femoral
*Parsvakonasana* (Extended side angle)	Forearm supination	Biceps brachii	Musculocutaneous
		Supinator	Radial
	Knee flexion	Hamstrings	Sciatic
*Chaturanga* (4‐limbed staff)	Scapula protraction	Serratus anterior	Long thoracic
	Scapula retraction	Rhomboids	Dorsal scapular
*Urdhva mukha svanasana* (Upward facing dog)	Elbow extension	Triceps brachii	Radial
	Ankle plantarflexion	Gastrocnemius and soleus	Tibial
*Adho mukha svanasana* (Downward facing dog)	Finger abduction	Dorsal interossei	Ulnar
	Ankle dorsiflexion	Tibialis anterior	Deep fibular
*Vrksasana* (Tree)	Elbow flexion	Brachialis	Musculocutaneous
	Wrist extension	Extensor carpi radialis longus and brevis	Radial
	Raised lower limb		
	Hip external rotation and flexion, knee flexion	Sartorius	Femoral
	Standing lower limb		
	Pelvis stabilization	Gluteus medius & minimus	Superior gluteal
	Hip adduction	Adductor magnus & longus	Obturator
	Hip extension	Gluteus maximus	Inferior gluteal
	Knee extension	Quadriceps femoris	Femoral
*Garudasana* (Eagle)	Shoulder adduction	Pectoralis major	Medial and lateral pectoral
	Hip adduction	Adductor muscles and gracilis	Obturator
*Virabhadrasana III* (Warrior III)	Shoulder extension	Latissimus dorsi	Thoracodorsal
		Teres major	Lower subscapular
	Hip extension	Gluteus maximus	Inferior gluteal
		Hamstrings	Sciatic
*Vasisthasana* (Side plank)	Trunk lateral flexion	Ipsilateral quadratus lumborum	Lumbar ventral rami
	Shoulder abduction	Deltoid	Axillary
*Navasana* (Boat)	Shoulder flexion	Pectoralis major	Medial and lateral pectoral
		Deltoid (anterior)	Axillary
	Finger extension	Extensor digitorum	Radial
	Trunk flexion	Rectus abdominis	Ventral rami T7–T12
	Hip flexion	Iliopsoas	Femoral

### The spinal and peripheral nerve workshop

**FIGURE 3:**
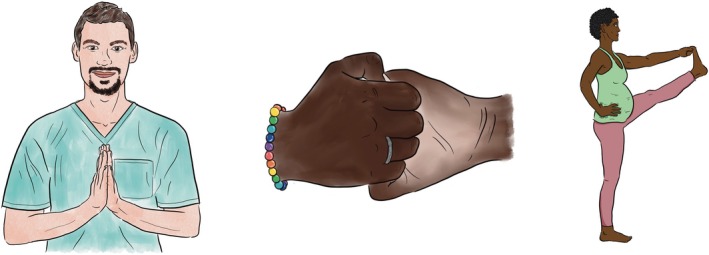
A review of key myotomes is demonstrated using various *mudras* and *asanas*. Left panel: *Anjali mudra* (salutation seal), C6: Elbow flexion, wrist extension; Middle panel: *Ganesha mudra* (remover of obstacles seal), C8: Finger flexion; Right panel: *Utthita hasta padangusthasana* (extended hand to big toe pose), L2: Hip flexion, L3: Knee extension, L4: Ankle dorsiflexion, L5: Big toe extension.

The Spinal and Peripheral Nerve Workshop reviews the brachial plexus, lumbosacral plexus, myotomes, and dermatomes. Myotomes are demonstrated using various *mudras* and *asanas* (Figure 3). For example, *Anjali mudra* (salutation seal) is used to demonstrate the C6 myotome for elbow flexion and wrist extension, and *Ganesha mudra (*remover of obstacles seal) is used to demonstrate the C8 myotome of finger flexion. *Utthita hasta padangusthasana* (extended hand to big toe pose) is used to demonstrate the myotomes for hip flexion (L2), knee extension (L3), ankle dorsiflexion (L4), and big toe extension (L5). After review of the myotomes, the attendees are led through a series of *asanas*. The *asanas* included in this workshop highlight the relevant muscles, their actions, and innervations via peripheral nerves, as well as via spinal nerves (myotomes). For instance, iliopsoas, the femoral nerve, and the L2 myotome are highlighted during hip flexion in *Vrksasana* (tree pose) (Supplement S1). After teaching the individual *asanas* and linking them together in a *vinyasa*, the attendees are asked to recall the muscles, their associated actions, and their innervation. Next, a more advanced sun salutation (*Surya Namaskar B*) is taught, focusing on sensory innervation via spinal (dermatome) and peripheral nerves.

### The neuroanatomy and neurological exam workshop

The Neuroanatomy and Neurological Exam Workshop reviews key neural tracts (corticospinal, spinothalamic, dorsal column medial lemniscus), the visual pathway, upper and lower motor neuron facial weakness, internuclear ophthalmoplegia, sensory versus cerebellar ataxia, and other neuroanatomical concepts. The included *asanas* highlight relevant content, such as the 12 cranial nerves [CN]. For example, *Utkata konasana* (goddess pose) was chosen to mimic the visual pathway and to explain visual deficits (Supplement S2). *Simhasana* (lion pose) is a yoga pose that mimics a lion's roar by sticking out the tongue. This *asana* was included to align with testing the hypoglossal nerve [CN XII] (Supplement S3). Common *mudras* mimic one of the tests for dysmetria in the coordination exam (Supplement S4). The yoga workshop progresses through all parts of the neurological exam in sequence. Consistent with the other two workshops, this workshop is interactive; beyond answering questions about muscles, actions, and innervations, attendees are encouraged to explain selected clinical concepts.

### Instructor versions of the workshops

The instructor's version of each workshop includes step‐by‐step directions on how to get into and out of a pose. It also specifies which limb to move, as it is important for the instructor to “mirror” the students when facing them (i.e., when a yoga instructor is facing the class, they should say *left* foot when they move their own *right* foot). When appropriate, the instructor version also contains suggestions for pose modifications. Finally, background notes, particularly on clinical concepts, are embedded within the instructor version. The instructor notes are not shared with the attendees, as they were created to aid the educator who may not be a certified yoga instructor.

### Skill level of the participants

Previous yoga experience is not necessary to participate in the sessions. The included *asanas* are fundamental poses taught in a standard yoga class. Furthermore, at the outset of each workshop, the facilitators explain that yoga is not a performance, nor a competition, but rather a journey of self‐discovery and connection. Accordingly, there is no expectation nor pressure for participants to complete every pose. Individuals are encouraged to focus on their own practice, emphasizing breath, body, mind, and movement.

### Sanskrit, Latin, Greek, and anatomy common language^19^

**FIGURE 4:**
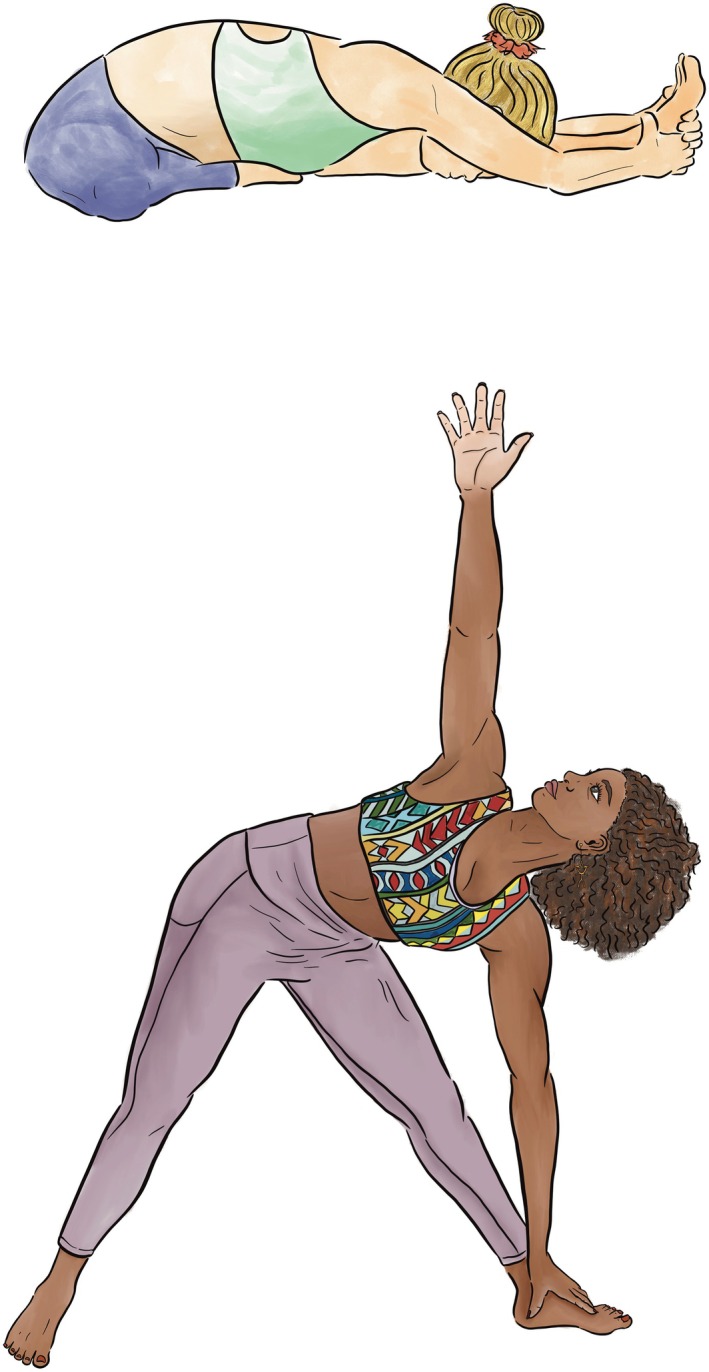
The Sanskrit names of various *asanas* reflect common anatomical and clinical terms. *Janu* translates as knee or bend and is seen in the genu of the corpus callosum or in genicular arteries; *Kona* translates as angle and is seen in the word gonioscope. Top panel: *Janu sirsasana* (head‐to‐knee pose); Bottom panel: *Trikonasana* (triangle pose).

Learning anatomical and medical terminology is akin to learning a new language, and understanding the etymology can greatly aid in this learning. Throughout the workshops, connections are made between the Sanskrit names of the *asanas*, the Latin or Greek roots, the medical or anatomical terms, and common English words. For example, the word ‘yoga’ comes from the Sanskrit word *yuj*, which means ‘union’. This is the same root for the English word “yoked,” which is often used to describe how the oculomotor nerve [CN III] and the abducens nerve [CN VI] work in unison to accomplish conjugate lateral gaze. Another example is the Sanskrit word *nava*, which translates to ‘boat’ in English and appears in *navasana* (boat pose). This is the same root seen in the anatomical word navicular, a boat‐shaped bone in the foot, as well as the word navy, which refers to military operations by boat. Furthermore, the Sanskrit word *janu* (knee or bend) appears in *janu sirsasana* (head‐to‐knee pose). In Latin, the word for knee is *genu*; the genu of the corpus callosum, the genu of the internal capsule, and the genicular arteries all refer to the knee or a bend. This root is also in the word genuflect, which means to bend the knee in worship as a sign of respect. Lastly, *trikonasana* translates to triangle pose. The Sanskrit term *kona*, meaning angle, appears in the names of many *asanas*. Similarly, the Greek root *gony* denotes an angle; for example, a gonioscope measures the angle between the iris and cornea, while a goniometer is used in physical therapy to measure joint angle. Thus, these examples illustrate how Sanskrit, Latin, and Greek roots underpin many anatomical and clinical terms (Figure 4).

### Pilot study

This study was classified as exempt by the Institutional Review Board at the University of California, San Francisco (UCSF) (Dec 2, 2019; IRB # 19‐28436). All workshop participants completed a graduate anatomy course as a part of their health professions program prior to the yoga session. The UCSF campus is a graduate health sciences campus with students in the Schools of Pharmacy, Dentistry, Medicine, Nursing, and Physical Therapy. As musculoskeletal anatomy is a significant component of medical and physical therapy student coursework, but is not emphasized in pharmacy, dental, nor nursing school curricula, medical and physical therapy students were recruited for the preliminary studies.

### Methodology

First‐year and second‐year medical students (*n* = 47), and second‐year physical therapy students (*n* = 94) who had completed a musculoskeletal anatomy course were recruited. Medical students' participation was voluntary, as this cohort of students had several weeks within the curriculum dedicated to wellness activities. Opportunities to participate were announced a few weeks in advance, allowing students to register, and each yoga workshop filled up within 1 day of the announcement. Participation of physical therapy students was mandatory, and the workshops were integrated into their required coursework. Due to time constraints, the workshop was only offered once per academic year for each student cohort. Pretest assessments were distributed at the beginning of each workshop, followed by completion of the posttest and wellness survey after the yoga session. Sample questions from the pretest are included (Supplement S5). A nonparametric Wilcoxon Signed‐Rank test was used to compare pretest and posttest scores. After filling out a one‐question wellness survey, the participants were asked to fill out a workshop evaluation survey with open‐ended questions, which asked them to comment on the strengths, weaknesses, influence on well‐being, likelihood of starting a yoga practice, and suggestions for improvement.

The Neuroanatomy Workshop was a later addition to this series, and therefore it was not included in the pilot study.

### Results

In all cohorts, the subjects' test scores demonstrated statistically significant improvement (*p* ≤ 0.05) between the pretest and posttest, while no significant differences were identified on the two control questions (Table 2). We did not offer any workshops in 2020 due to the COVID‐19 pandemic. We continued to offer these workshops in 2024 and 2025, but data collection ceased after 2023 because the workshops were uniformly positive and statistically significant across every cohort.

**TABLE 2:** Pretest and posttest assessment data by workshop.

Year	Cohort	Workshop	*n*	Participants	Average	Average	Test questions	Control questions
					Pretest scores	Posttest scores	*p*‐value	*p*‐value
2019	1	Musculoskeletal	23	MS1s	6.9/10 (SD 1.9)	8.4/10 (SD 1.2)	<0.001	0.096
2021	2	Musculoskeletal	24	MS1s & MS2s	7.4/10 (SD 2.2)	8.6/10 (SD 1.2)	0.02	0.81
2022	3	Musculoskeletal	47	PT2s	7.2/10 (SD 1.8)	9/10 (SD 0.9)	<0.001	0.51
2023	4	Spinal & peripheral nerves	47	PT2s	6.5/10 (SD 1.9)	8.8/10 (SD 1.2)	<0.001	0.06

Abbreviations: MS1, 1st year medical student; MS2, 2nd year medical student; PT2, 2nd year physical therapy student.

The wellness question “Did this session contribute to a sense of wellness?” was rated on a 1–5 Likert scale (1 = Poor and 5 = Excellent). The mean score across all cohorts was 4.7 out of 5 (*n* = 141).

The comments from the evaluation survey were overwhelmingly positive, complementing the high score of 4.7 out of 5 on the wellness question. The attendees appreciated the duality of movement tied to learning and noted that it was accessible to those without prior yoga experience and promoted wellness. The most common constructive criticism was that some attendees would have liked to have light background meditation music. There were also comments requesting more time to hold the poses, and physical therapy students wanted to highlight concentric versus eccentric muscle contractions. A representative selection of comments from the different student cohorts is outlined in Table 3. Comments about weaknesses overlapped those regarding suggestions for improvement and thus are grouped together.

**TABLE 3:** Comments from the evaluation survey.

Theme	Sample quotes
Strengths: fun	*Great pace, relaxing, educational, but fun!* *Engaging, fun, interesting!* *This was a beautiful experiential moment* *I loved it!* *Fun way to visualize muscle actions* *Fun and effective way to connect anatomy with function*
Strengths: kinesthetic learning	*I am a kinesthetic learner, and this helped a lot—as well as the repetition* *Very helpful for kinesthetic learner. Easy to understand and follow even for doing yoga for the first time* *I am a kinesthetic learner and found this session VERY helpful* *Kinesthetic learning opportunity* *It was a great and kinesthetic way to learn*
Strengths: anatomy	*Awesome to learn while moving!* *Loved the dual taste of yoga and cognitive task* *I believe this was an excellent review of anatomy while still promoting health and wellness* *I think this was great for learning myotomes* *Very engaging application of anatomy* *Creative integration of wellness and anatomy review* *I enjoyed calling out the names of muscles used, nerve innervation to practice while in the pose*
Strengths: yoga teaching	*I liked the pace and openness to alternative positions for those of us who are unable to assume the correct positions* *I appreciated the walk through for poses and emphasis on breathing* *Good pacing* *Clarity of instruction and welcome space* *Flow made sense and was appropriate; good choice of poses* *Clear, well‐explained* *As someone who doesn't practice yoga, I thought the instructions were very clear*
Well‐being	*I believe this was an excellent review of anatomy while still promoting health and wellness*. *Feel very balanced and clear‐headed afterwards* *This was very calming and relaxing* *I feel very rejuvenated, both physically and mentally* *I'm feeling calm and peaceful* *Helped me relax and de‐stress* *I feel like melted butter* 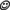 *I feel loose, educated, and zen!*
Likelihood of starting a yoga practice	*I have previously done yoga, but this encouraged me to practice more* *This has made me want to do more yoga in the future* *I realized how inflexible I am* *I want to get back into yoga* *I used to practice regularly but haven't for a few years, thus did reignite my interest in yoga* *Already do yoga*
Weaknesses/suggestions for improvement	*Getting tested was a bit stressful* *More opportunity for flow* *More corpse pose* *Could be more in‐depth in higher level courses* *Room was not spacious enough* *More meditation at the end* *Maybe some ambient sound during cool down* *Longer corpse pose* *Add contraction type (concentric versus eccentric)* *Let's do it more often* *Spend more time breathing in poses*

## DISCUSSION

Consistent with previous studies,^4^, ^5^, ^6^, ^7^, ^8^, ^9^, ^10^ anatomy content was presented during the yoga workshops. These prior studies, as well as the current workshops, provided a unique way for students to learn anatomy through a wellness activity. Similar to previous research highlighting the effectiveness of kinesthetic learning on student outcomes, integrating yoga into anatomy education offers students an additional kinesthetic learning approach.^11^, ^12^ There were, however, some ways that the previous studies differed from the current workshop. In the present study, nearly all regions of the body were engaged (head, neck, abdomen, back, upper limbs, lower limbs), while previous studies mainly focused on 1–2 regions.^4^, ^5^, ^6^, ^7^ Additionally, previous yoga anatomy studies^4^, ^5^, ^6^, ^7^, ^8^, ^9^, ^10^ focused solely on the musculoskeletal system, whereas these workshops also included peripheral and spinal nerves, and clinically relevant neuroanatomy, making them amenable to undergraduate and graduate health professions education curricula. Anatomical and clinical terms were supported by learning the etymology of many yoga *asanas*. A subtle distinction between the current study and some of the previous studies was that in the present study, the teaching of anatomy was concurrent with the movements of getting into and holding yoga poses, thereby fully engaging both body and mind, whereas prior studies separated yoga movements from the anatomy instruction.^4^, ^7^ These workshops also included many spiritual components of yoga, such as the meaning of *mudras*, and the meaning of “*Om*,” creating a holistic experience, which is unique when compared with earlier yoga anatomy research.^4^, ^5^, ^6^, ^7^, ^8^, ^9^, ^10^ These studies also assessed the impact of yoga on well‐being with positive results.

### Limitations

Demographic data were not obtained and therefore were not available for further analysis. However, this would be beneficial information to collect in future studies.

Results from the wellness survey and workshop evaluation comments indicated a strong appreciation for the wellness component of this activity. However, while these workshops were mandatory for the physical therapy students, they were voluntary for the medical student participants; therefore, it is possible that the medical students who were included in the study already had a greater focus on their personal wellness.

The two “control” questions on the pretest and posttest assessed content from the broader anatomy curriculum which was not specifically covered during the yoga intervention. To better assess learners' knowledge reinforcement, the posttest should be repeated at a later time, as opposed to directly after the yoga intervention, as people often do well immediately following an intervention, reflecting their short‐term recall. Thus, future studies should explore including additional assessments following a yoga anatomy session to address reinforcement and retention of anatomy knowledge. Additionally, subsequent research should also consider a randomized control design in which the intervention group participates in these yoga workshops, while the control group engages in an anatomy review without integrated physical movement, thereby analyzing knowledge retention and wellness outcomes between the two groups.

The artwork was created specifically for these workshops by two different medical student artists. The image size and background tone were variable. Checking that materials meet the Web Content Accessibility Guidelines (WCAG) would help to align with the Americans with Disabilities Act (ADA) mandates regarding accessibility.

While the creator (DR) of these workshops is a certified yoga instructor, a yoga certification is not required to teach these workshops. All of the workshops included teachers who were not certified yoga instructors. With the detailed instructions and modifications noted in the teacher's versions of the workshops, they were able to guide participants safely into and out of the poses. If one does not have the knowledge of Sanskrit, mudras, or some of the holistic aspects of yoga, these workshops can be tailored with a focus just on the anatomy. It should be noted, however, that yoga instructors are trained to observe the participants at all times to ensure their safety and to give brief cues if adjustments are warranted. As in any physical activity, safety practices and liability are a concern, so ensuring that these are in place is recommended. Replicable research and repeatable methods may be affected if there is no certified yoga teacher present.

### Challenges

These workshops were first created when the medical students at UCSF had dedicated in‐person wellness time reserved in the curriculum, during which these workshops filled up very quickly. During the COVID‐19 pandemic, the curriculum became remote, which did not lend itself well to facilitating these workshops. After the pandemic, the dedicated in‐person wellness sessions no longer existed due to changes in the School of Medicine curriculum. Without dedicated time reserved for wellness, it became challenging to find time to offer these workshops, as health professions education curricula for students and residents are densely packed. In addition, room availability was challenging, as many lecture halls have fixed tables and chairs. Thus, identifying and scheduling a room that accommodates more than 20 attendees (with their mats) was critical.

### Versatility

These workshops can be easily adapted for the specific learner, from students in undergraduate anatomy courses to medical and physical therapy students in their preclinical curriculum to residents in various specialties. Due to their modular nature, one can “mix and match” content from the three workshops if desired. In addition, these workshops can be taught with very little (to no) cost for the instructors, making them a cost‐effective method for anatomy instruction. It is, however, recommended to have a supply of yoga mats for attendees who do not have their own.

### Next steps

We are currently collaborating with one of the artists to standardize image resolution, background tone, and sizing across all paintings and drawings presented in these workshops. Additionally, we are currently designing a yoga workshop focused on pelvic anatomy.

Moving forward, we plan to incorporate some of the feedback from the evaluation survey, including background meditation music, holding poses for longer, and highlighting concentric versus eccentric muscle contractions specifically for physical therapy students.

The Spinal and Peripheral Nerves workshop was recently taught to over 100 attendees at a physical therapy conference (February 2025).^20^ We plan to continue teaching these workshops at future anatomical, allied health sciences, or clinical conferences.

The Spinal and Peripheral Nerves workshop was also taught to anesthesia residents, but they did not complete the pretest, posttest, or wellness survey due to time constraints. Their workshop was voluntary and was scheduled at the end of a long education day. We plan to offer our workshops to some of our institution's other residency programs, such as orthopedics and neurology.

In future studies, we can examine the impact of these yoga anatomy workshops on students' anatomical knowledge through randomized control and experimental groups, as well as through pretests, posttests, and retention tests (posttests conducted at later intervals) to determine if teaching anatomy through yoga fosters a better understanding and retention of the anatomy taught in their prior courses.

### Conclusions

These workshops can be implemented at other teaching institutions to help foster anatomy education and well‐being. Of note, the workshops can be tailored to the specific needs of the audience.

Based on the preliminary workshops, participants demonstrated improved anatomy knowledge and reported a positive sense of overall wellness. Experiential learning theory suggests that when one is physically engaged in an activity, they are engaged with the material, which reduces their cognitive load.^16^ Holistic education focuses on the whole person and integrates the learner with the curriculum.^14^, ^15^ It comprises embodiment (knowing through our bodies), enactment, and embedding in the culture.^17^ This approach highlights the interconnectedness of the mind and body.^15^ By practicing yoga *asanas*, *vinyasas*, and sun salutations, while reviewing relevant anatomy and simultaneously linking the anatomy to Sanskrit, *mudras*, *Om*, and other elements of an authentic yoga practice, these yoga workshops exemplify a holistic and embodied approach to learning.

## AUTHOR CONTRIBUTIONS

**Dana Rohde:** Conceptualization; investigation; funding acquisition; writing – original draft; methodology; validation; visualization; writing – review and editing; formal analysis; project administration; data curation; resources. **Madeleine E. Norris:** Investigation; writing – review and editing. **Jennifer R. Kinder:** Investigation; writing – review and editing. **Barbie Klein:** Investigation; writing – review and editing. **Derek Harmon:** Investigation; writing – review and editing.

## FUNDING INFORMATION

This work was supported by the Sexton Sutherland Endowed Chair in Human Anatomy, Academy of Medical Educators, University of California, San Francisco.

## CONFLICT OF INTEREST STATEMENT

The authors declare that they have no conflict of interest.

## ETHICS STATEMENT

Exempt Certification by UCSF's institutional review board. Dec 2, 2019. IRB # 19‐28436.

## Supporting information


Supplement S1.

Supplement S2.

Supplement S3.

Supplement S4.

Supplement S5.
