# Supplementary figures and images for: Integrating yoga into anatomy and clinical medicine education: A holistic approach to learning
Source: Anat Sci Educ. 2026 Mar 19;19(7):1097–109. doi: 10.1002/ase.70225 (PMC13332515; doi:10.1002/ase.70225)

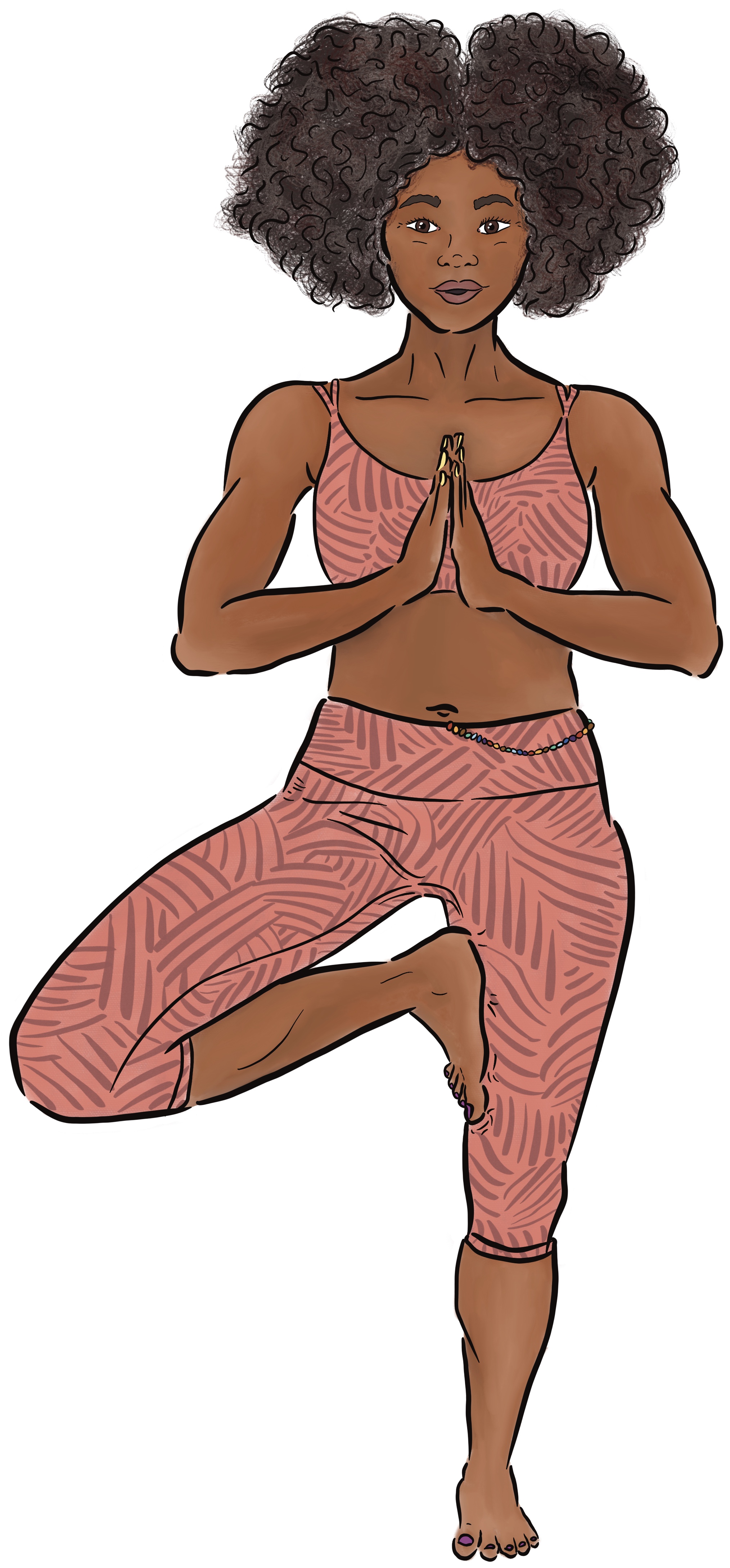

Supplement: Supplementary file 1 — Supplement S1. [file ASE-19-1097-s001.tiff]

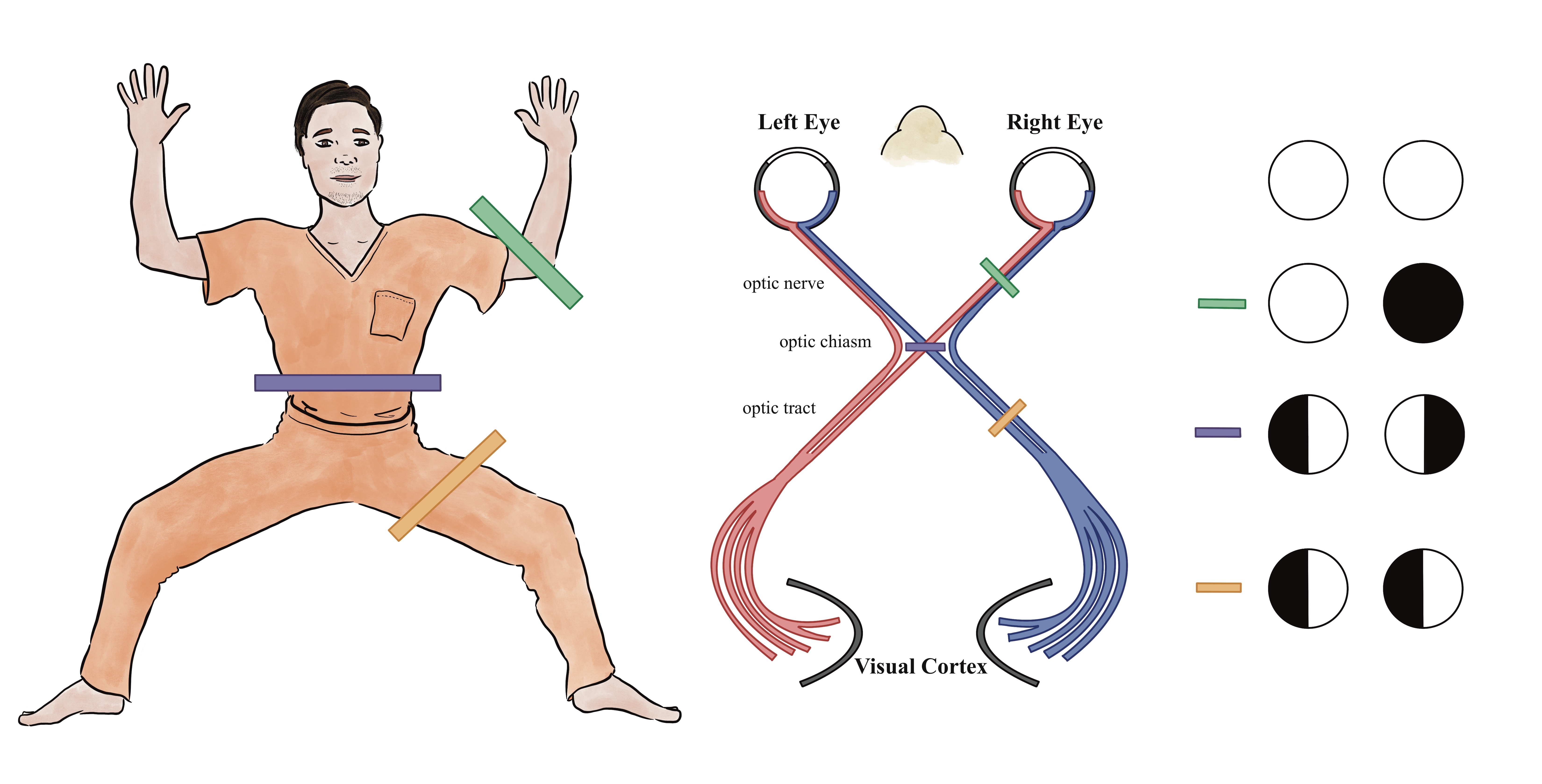

Supplement: Supplementary file 2 — Supplement S2. [file ASE-19-1097-s005.tiff]

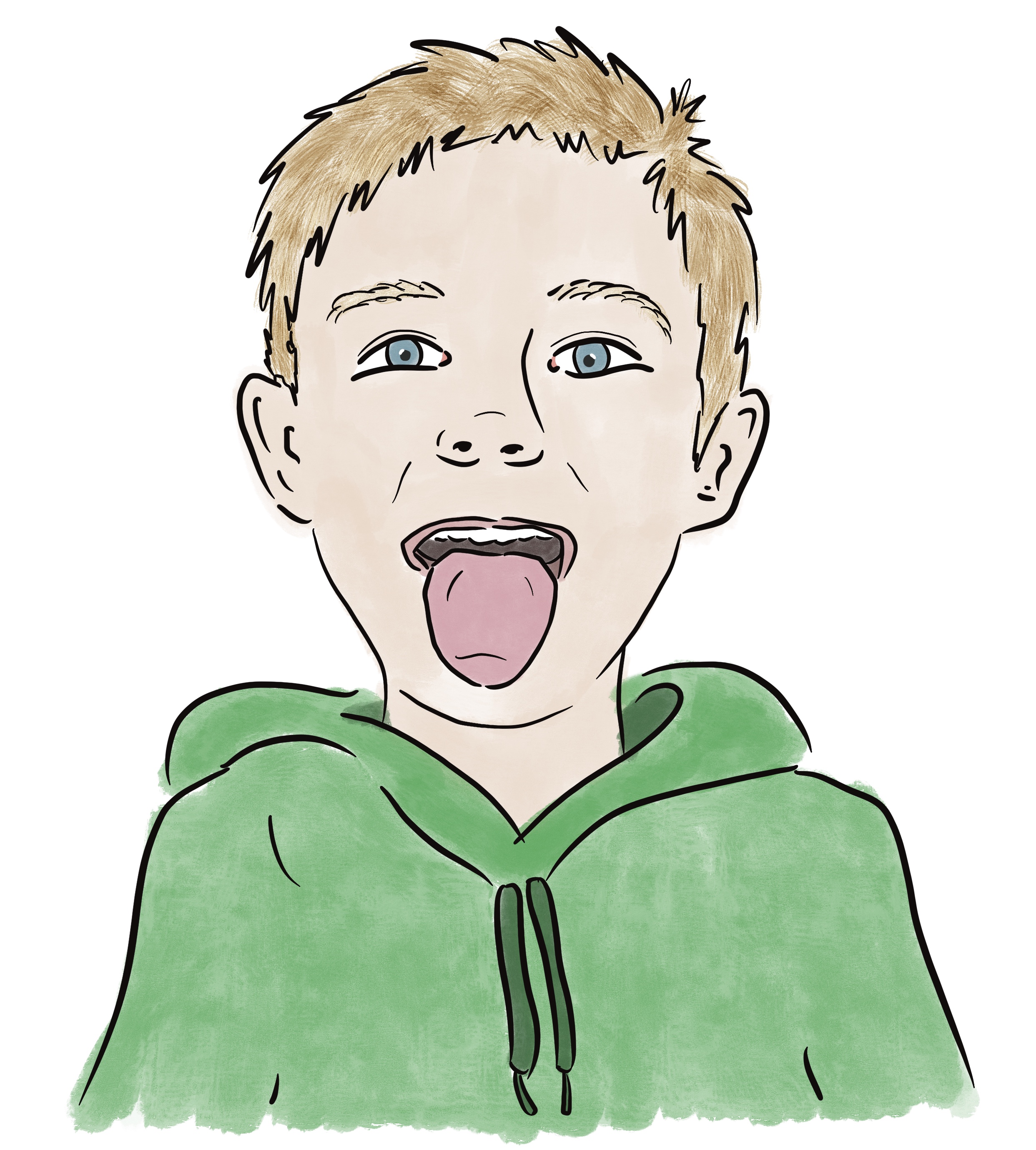

Supplement: Supplementary file 3 — Supplement S3. [file ASE-19-1097-s004.tiff]

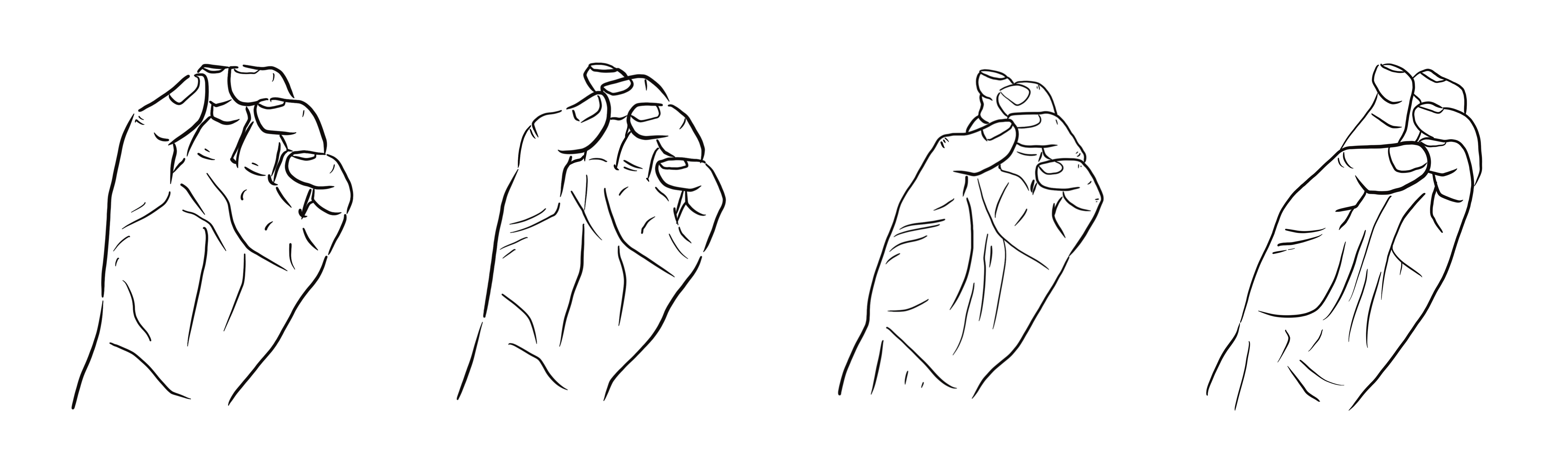

Supplement: Supplementary file 4 — Supplement S4. [file ASE-19-1097-s003.tiff]

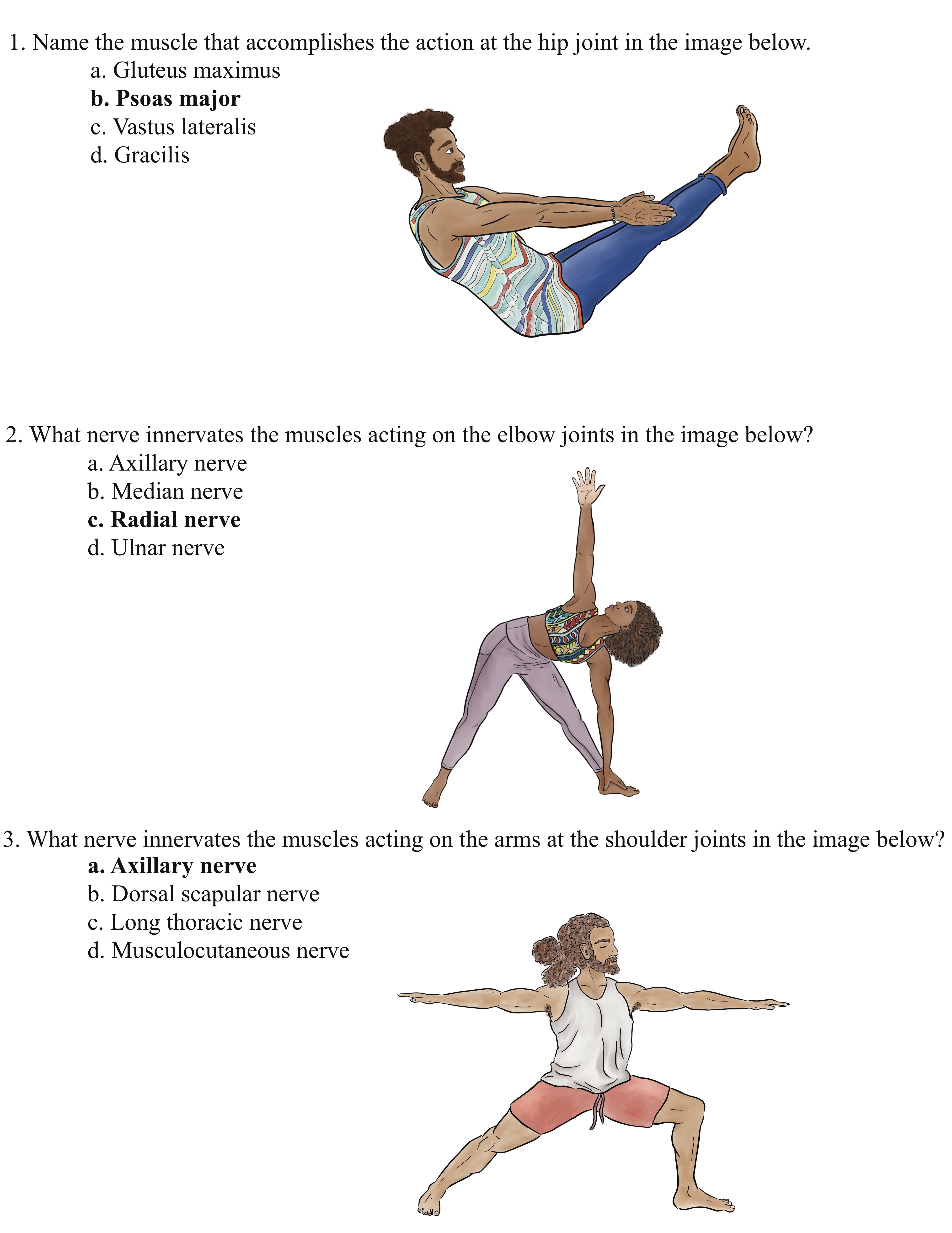

Supplement: Supplementary file 5 — Supplement S5. [file ASE-19-1097-s002.tiff]
